# Rehabilitation for Musculoskeletal Disorders: The Emergence of Serious Games and the Promise of Personalized Versions Using Artificial Intelligence

**DOI:** 10.3390/jcm12165310

**Published:** 2023-08-15

**Authors:** Julien Favre, Alexis Cantaloube, Brigitte M. Jolles

**Affiliations:** 1 Swiss BioMotion Lab, Lausanne University Hospital, University of Lausanne (CHUV-UNIL), CH-1011 Lausanne, Switzerland; 2 The Sense Innovation and Research Center, CH-1007 Lausanne, Switzerland; 3 Institute of Electrical and Micro Engineering, Ecole Polytechnique Fédérale Lausanne (EPFL), CH-1015 Lausanne, Switzerland

According to the World Health Organization (WHO), musculoskeletal conditions are among the most common health problems, affecting approximately 1.71 billion people worldwide [1]. These disorders of the joints, ligaments, muscles, nerves, and tendons among others supporting structures of the body are the leading cause for rehabilitation prescription. Indeed, they account for about two thirds of adult rehabilitation and are one of the most common causes of rehabilitation provided to children [1]. If rehabilitation is so present in the treatment of musculoskeletal conditions, it is because it covers a range of important objectives, including restoring, maintaining, and improving physical function and mobility, as well as relieving pain and improving quality of life.

During the last few years, there has been a remarkable intake of technology in musculoskeletal rehabilitation, notably with the development of serious games and their transfer to the clinic. Serious games in the case of musculoskeletal rehabilitation generally consist of systems that measure how the patients move and provide them with guidance to execute specific movements in a playful manner [2]. They assist the clinicians for certain exercises, especially by allowing the integration of quantitative movement data into rehabilitation, reducing the need for constant supervision, and enhancing patients’ adherence [3]. This has the potential to improve clinical outcomes, reduce costs and facilitate accessibility to rehabilitation programs [4]. Serious games have already been proposed for a wide range of applications to restore normal movement patterns, improve balance, proprioception, and mobility, as well as develop muscular strength, and address specific gait abnormalities [5,6]. They are built on three technological bricks, as described in the following paragraphs.

Movement measurement is the first key component of serious games. It provides an objective biomechanical description of the movements executed, both in terms of quality and quantity. Cameras, inertial measurement units (IMUs), as well as electromyographic (EMG) and force sensors are the most commonly used devices to measure human movement in serious games [7,8]. Once processed, the recordings from these devices can provide detailed information on the range of motion, number of repetitions and technique [9], muscle activity [7], as well as on the trajectory of the center of pressure [10], among many other possible biomechanical parameters. Such quantitative data on movement execution enable the clinicians to gain insights into the impairments specific to each patient and optimize treatment strategies. They can also be used to guide the patients by providing feedback on movement executions. For example, Chan et al. [11] showed that novice runners can modify their vertical ground-reaction force pattern using an instrumented treadmill with real-time display of the force signal and that this modification can prevent injury.

With serious games, the biofeedback is brought to another level due to extended reality (XR), the second major technological component of serious games. XR is a general term covering virtual reality (VR), augmented reality (AR), and mixed reality (MR). All these types of extended realities have been used in musculoskeletal rehabilitation, particularly to provide more advanced instructions and feedback to the patients and place them in immersive and interactive environments. XR plays an important role in the communication with the patients, particularly for complex exercises and movements that should be executed precisely. A variety of devices can be used to deliver XR. Nevertheless, a display in the form of a screen, tablet, or headset is often considered because of its intuitive nature [9]. In this case, avatars or graphics are frequently used to guide patient movements. For example, to train head mobility in patients with chronic neck pain, Sarig Bahat et al. [12] used a headset to place the patients in a VR sky where they controlled an airplane by moving their head. Comprehensive and intuitive visual feedback has been shown to be beneficial for motor learning and promote engagement [9]. Similarly, sounds can be used, on their own or coupled with a display, to deliver auditory cues, instructions, or feedback [13,14]. Multi-sensory experiences, notably including haptics, are also possible [15,16]. Moreover, performance metrics, which provide quantitative data on parameters such as speed, accuracy, and the number of repetitions, are often included to help the patients track their progress.

Gamification is the third fundamental technological component of serious games. It consists of adding game aspects into the rehabilitation process, therefore improving motivation and involvement, which are critical for treatment success [2,17,18]. Gamification can be seen as a rehabilitation strategy where exercises and activities are transformed into interactive games designed to improve participation and enjoyment while keeping the focus on the therapeutic goals. To create a more immersive and goal-oriented experience, serious games incorporate aspects such as challenges, prizes, progress tracking, and competition. For example, in the case of total knee arthroplasty rehabilitation, Qiu et al. [19] proposed a fishing serious game where, in addition to controlling the catching of the fishes with the knee flexion angle, elements demanding decision making, such as selecting the baits and solving puzzles, as well as rewards based on the caught fish attributes, have been included.

Serious games have been booming lately, with highly promising results for a variety of musculoskeletal conditions. For instance, they have been shown to be effective in joint arthroplasty rehabilitation [20,21,22,23], as well as in the treatment of chronic neck pain and shoulder impingement syndrome [24]. Furthermore, pain reductions have been reported in patients with acute burns [25,26], and in patients with chronic musculoskeletal disorders [27,28]. Encouraging results were also obtained regarding sport injury prevention [29]. In addition, reduced anxiety and stress, as well as increased enjoyment during physical rehabilitation and physiotherapy were observed with serious games [26]. Of course, the efficacy generally reported for serious games so far should be considered critically. Serious games is an emerging field and further assessment of more completed solutions on larger populations will be needed to be more specific on the advantages and drawbacks. For example, multiple studies reported no significant differences in clinical outcomes between serious games and traditional exercises, but nonetheless considered the instrumentation to be beneficial [21,24,25,28]. In these cases, the conclusions were mostly driven by the increase in the sense of engagement [3] and the possibility to deliver high-quality care at lower costs, contributing to more accessible and efficient healthcare [4]. While this editorial focuses on musculoskeletal rehabilitation, it is important to mention that serious games for other medical conditions have been proposed with comparable positive results and appreciations, notably for stroke [30], Parkinson’s disease [31], and spinal cord injury [32].

Following the engaging state-ot-the-art advancements presented above, a limitation, or rather a perspective, should be highlighted. While current serious games for musculoskeletal conditions can be adapted by clinicians to the individual needs of the patients, there is a lack of support in doing so. In fact, currently, the adaptations are mostly based on the clinicians’ expertise. Similarly to the trend observed in other medical applications [33,34,35], adding a data science component to serious games could fully unlock their potential by allowing personalized treatment plans based on the therapeutic trajectories observed with prior patients. To address this challenge, it will be necessary to build large datasets with patient characteristics and progression, and to develop mathematical models that are able to suggest personalized rehabilitation programs. This look into the future might not be that distant, as serious games can already store relevant data about the patients and their rehabilitation journey, and artificial intelligence (AI) algorithms are capable of processing large amounts of data to identify patterns and hopefully soon recommend adaptations to the serious games based on the individual situation of each patient.

In conclusion, serious games are extremely promising for the rehabilitation of musculoskeletal conditions and their value should significantly increase in the future with the incorporation of AI to suggest personalized therapeutic objectives and individualize the games. In view of the importance of musculoskeletal rehabilitation worldwide, serious games could play a major role in improving global health and lower the societal and economic burden of musculoskeletal disorders. Finally, it is worth mentioning that serious games can be deployed at home [4] and that newer technologies for movement measurement and XR [36,37] will facilitate such implementation in the future. As time progresses, we will be more exposed to these newer technologies in our daily life, which should also contribute to better acceptance and clinical outcomes of rehabilitation using serious games.

## References

[1] Cieza A., Causey K., Kamenov K., Hanson S.W., Chatterji S., Vos T. (2020). Global estimates of the need for rehabilitation based on the Global Burden of Disease study 2019: A systematic analysis for the Global Burden of Disease Study 2019. Lancet.

[2] Alfieri F.M., Da Silva Dias C., De Oliveira N.C., Battistella L.R. (2022). Gamification in Musculoskeletal Rehabilitation. Curr. Rev. Musculoskelet. Med..

[3] Liberatore M.J., Wagner W.P. (2021). Virtual, mixed, and augmented reality: A systematic review for immersive systems research. Virtual Real..

[4] Berton A., Longo U.G., Candela V., Fioravanti S., Giannone L., Arcangeli V., Alciati V., Berton C., Facchinetti G., Marchetti A. (2020). Virtual Reality, Augmented Reality, Gamification, and Telerehabilitation: Psychological Impact on Orthopedic Patients’ Rehabilitation. J. Clin. Med..

[5] Howard M.C. (2017). A meta-analysis and systematic literature review of virtual reality rehabilitation programs. Comput. Hum. Behav..

[6] Li L., Yu F., Shi D., Shi J., Tian Z., Yang J., Wang X., Jiang Q. (2017). Application of virtual reality technology in clinical medicine. Am. J. Transl. Res..

[7] Giggins O.M., Persson U., Caulfield B. (2013). Biofeedback in rehabilitation. J. NeuroEngineering Rehabil..

[8] Verbrugghe J., Knippenberg E., Palmaers S., Matheve T., Smeets W., Feys P., Spooren A., Timmermans A. (2018). Motion detection supported exercise therapy in musculoskeletal disorders: A systematic review. Eur. J. Phys. Rehabil. Med..

[9] Brennan L., Dorronzoro Zubiete E., Caulfield B. (2019). Feedback Design in Targeted Exercise Digital Biofeedback Systems for Home Rehabilitation: A Scoping Review. Sensors.

[10] Vogt S., Skjæret-Maroni N., Neuhaus D., Baumeister J. (2019). Virtual reality interventions for balance prevention and rehabilitation after musculoskeletal lower limb impairments in young up to middle-aged adults: A comprehensive review on used technology, balance outcome measures and observed effects. Int. J. Med. Inf..

[11] Chan Z.Y.S., Zhang J.H., Au I.P.H., An W.W., Shum G.L.K., Ng G.Y.F., Cheung R.T.H. (2018). Gait Retraining for the Reduction of Injury Occurrence in Novice Distance Runners: 1-Year Follow-up of a Randomized Controlled Trial. Am. J. Sports Med..

[12] Sarig Bahat H., Croft K., Carter C., Hoddinott A., Sprecher E., Treleaven J. (2018). Remote kinematic training for patients with chronic neck pain: A randomised controlled trial. Eur. Spine J..

[13] Reh J., Hwang T.-H., Schmitz G., Effenberg A. (2019). Dual Mode Gait Sonification for Rehabilitation After Unilateral Hip Arthroplasty. Brain Sci..

[14] Röhner E., Mayfarth A., Sternitzke C., Layher F., Scheidig A., Groß H.-M., Matziolis G., Böhle S., Sander K. (2021). Mobile Robot-Based Gait Training after Total Hip Arthroplasty (THA) Improves Walking in Biomechanical Gait Analysis. J. Clin. Med..

[15] Rose T., Nam C.S., Chen K.B. (2018). Immersion of virtual reality for rehabilitation-Review. Appl. Ergon..

[16] Escamilla-Nunez R., Michelini A., Andrysek J. (2020). Biofeedback Systems for Gait Rehabilitation of Individuals with Lower-Limb Amputation: A Systematic Review. Sensors.

[17] Maclean N., Pound P., Wolfe C., Rudd A. (2000). Qualitative analysis of stroke patients’ motivation for rehabilitation. BMJ.

[18] Allam A., Kostova Z., Nakamoto K., Schulz P.J. (2015). The Effect of Social Support Features and Gamification on a Web-Based Intervention for Rheumatoid Arthritis Patients: Randomized Controlled Trial. J. Med. Internet Res..

[19] Qiu Y., Li K.M., Neoh E.C., Zhang H., Khaw X.Y., Fan X., Miao C. Fun-Knee™: A novel smart knee sleeve for Total-Knee-Replacement rehabilitation with gamification. Proceedings of the 2017 IEEE 5th International Conference on Serious Games and Applications for Health (SeGAH).

[20] Blasco J., Igual-Camacho C., Blasco M., Antón-Antón V., Ortiz-Llueca L., Roig-Casasús S. (2021). The efficacy of virtual reality tools for total knee replacement rehabilitation: A systematic review. Physiother. Theory Pract..

[21] Byra J., Czernicki K. (2020). The Effectiveness of Virtual Reality Rehabilitation in Patients with Knee and Hip Osteoarthritis. J. Clin. Med..

[22] Gazendam A., Zhu M., Chang Y., Phillips S., Bhandari M. (2022). Virtual reality rehabilitation following total knee arthroplasty: A systematic review and meta-analysis of randomized controlled trials. Knee Surg. Sports Traumatol. Arthrosc..

[23] Peng L., Zeng Y., Wu Y., Si H., Shen B. (2021). Virtual reality-based rehabilitation in patients following total knee arthroplasty: A systematic review and meta-analysis of randomized controlled trials. Chin. Med. J..

[24] Gumaa M., Rehan Youssef A. (2019). Is Virtual Reality Effective in Orthopedic Rehabilitation? A Systematic Review and Meta-Analysis. Phys. Ther..

[25] Lin H.T., Li Y.I., Hu W.P., Huang C.C., Du Y.C. (2019). A Scoping Review of The Efficacy of Virtual Reality and Exergaming on Patients of Musculoskeletal System Disorder. J. Clin. Med..

[26] Scapin S., Echevarría-Guanilo M.E., Junior P.R.B.F., Gonçalves N., Rocha P.K., Coimbra R. (2018). Virtual Reality in the treatment of burn patients: A systematic review. Burns.

[27] Brea-Gómez B., Torres-Sánchez I., Ortiz-Rubio A., Calvache-Mateo A., Cabrera-Martos I., López-López L., Valenza M.C. (2021). Virtual Reality in the Treatment of Adults with Chronic Low Back Pain: A Systematic Review and Meta-Analysis of Randomized Clinical Trials. Int. J. Environ. Res. Public Health.

[28] Kantha P., Lin J.J., Hsu W.L. (2023). The Effects of Interactive Virtual Reality in Patients with Chronic Musculoskeletal Disorders: A Systematic Review and Meta-Analysis. Games Health J..

[29] Schuermans J., Van Hootegem A., Van den Bossche M., Van Gendt M., Witvrouw E., Wezenbeek E. (2022). Extended reality in musculoskeletal rehabilitation and injury prevention—A systematic review. Phys. Ther. Sport.

[30] Kim W.-S., Cho S., Ku J., Kim Y., Lee K., Hwang H.-J., Paik N.-J. (2020). Clinical Application of Virtual Reality for Upper Limb Motor Rehabilitation in Stroke: Review of Technologies and Clinical Evidence. J. Clin. Med..

[31] Lei C., Sunzi K., Dai F., Liu X., Wang Y., Zhang B., He L., Ju M. (2019). Effects of virtual reality rehabilitation training on gait and balance in patients with Parkinson’s disease: A systematic review. PLoS ONE.

[32] De Araújo A.V.L., Neiva J.F.d.O., Monteiro C.B.d.M., Magalhães F.H. (2019). Efficacy of virtual reality rehabilitation after spinal cord injury: A systematic review. BioMed Res. Int..

[33] Jie Z., Zhiying Z., Li L. (2021). A meta-analysis of Watson for Oncology in clinical application. Sci. Rep..

[34] (2019). Amisha; Malik, P.; Pathania, M.; Rathaur, V.K. Overview of artificial intelligence in medicine. J. Fam. Med. Prim. Care.

[35] Syrowatka A., Song W., Amato M.G., Foer D., Edrees H., Co Z., Kuznetsova M., Dulgarian S., Seger D.L., Simona A. (2022). Key use cases for artificial intelligence to reduce the frequency of adverse drug events: A scoping review. Lancet Digit. Health.

[36] Hellsten T., Karlsson J., Shamsuzzaman M., Pulkkis G. (2021). The Potential of Computer Vision-Based Marker-Less Human Motion Analysis for Rehabilitation. Rehabil. Process Outcome.

[37] Meulenberg C.J., de Bruin E.D., Marusic U. (2022). A perspective on implementation of technology-driven exergames for adults as telerehabilitation services. Front. Psychol..

